# Barriers and enablers to accessing support services offered by staff wellbeing hubs: A qualitative study

**DOI:** 10.3389/fpsyg.2022.1008913

**Published:** 2022-11-15

**Authors:** Chris Keyworth, Adnan Alzahrani, Lucy Pointon, Kerry Hinsby, Nigel Wainwright, Lucie Moores, Jenny Bates, Judith Johnson

**Affiliations:** ^1^School of Psychology, University of Leeds, Leeds, United Kingdom; ^2^Department of Basic Science, Prince Sultan bin Abdulaziz College for Emergency Medical Services, King Saud University, Riyadh, Saudi Arabia; ^3^Leeds and York Partnership Foundation Trust, Leeds, United Kingdom; ^4^Mid-Yorkshire Hospitals NHS Trust, Wakefield, United Kingdom; ^5^Bradford Royal Infirmary, Bradford Institute for Health Research, Bradford, United Kingdom; ^6^School of Public Health and Community Medicine, University of New South Wales, Kensington, NSW, Australia

**Keywords:** qualitative, healthcare professionals, mental health, wellbeing, healthcare workers

## Abstract

**Background:**

International efforts have been made to develop appropriate interventions to support the mental health needs of healthcare professionals in response to COVID-19. However, fewer staff have accessed these than expected, despite experiencing elevated levels of mental distress since the onset of the pandemic. Consequently, we aimed to examine the barriers and enablers for healthcare professionals in accessing interventions offered by a Staff Mental Health and Wellbeing Hub.

**Methods:**

Twenty-five semi-structured interviews were conducted with healthcare, social care and voluntary, community and social enterprise (VCSE) sector staff. Data were analysed using thematic analysis.

**Results:**

Four key themes were identified: (1) *Environment and Atmosphere in the Workplace*; (2) *The Impacts of COVID-19*; (3) *Confidentiality*; and (4) *Awareness and Communication of Resources*. Organisational environments were perceived as an important enabler of accessing the hub services for mental health and wellbeing support. This included the importance of recognising and responding to the ongoing pressures of COVID-19- specific challenges. Ensuring and communicating aspects of confidentiality, and ensuring clear and consistent communication of the benefits of the Hub may encourage help-seeking for mental health challenges among healthcare professionals.

**Discussion:**

Our findings highlight important considerations to increase uptake and engagement with services to support the mental health and wellbeing of healthcare professionals and associated staff and volunteers. Organisations aiming to increase employee uptake of these services should regularly circulate consistent and clear emails about what these services offer, provide training and information for managers so they can support staff to access these services and ensure access is confidential.

## Introduction

The mental health impacts of the COVID-19 pandemic on healthcare professionals have been significant (De Kock et al., 2021; Mitchell et al., 2022). During the early phases of the pandemic, the World Health Organisation outlined a series of mental health and psychosocial considerations aimed at healthcare professionals (World Health organisation, 2020). As part of this guidance, team leaders or managers in healthcare settings were instructed to ensure their staff were sufficiently informed of mental health services available to them. Despite this recognition, the healthcare professional workforce, including social care and voluntary sector staff (Aughterson et al., 2021; Blake et al., 2021) was placed under considerable strain as a direct impact of the pandemic. Internationally, the reasons for this have included: understaffing leading to burnout (the psychological syndrome characterised by emotional exhaustion and disengagement; Lasater et al., 2021), the fear of becoming infected (Liu et al., 2020), contact with death and suffering (Moreno-Jiménez et al., 2021) and a lack of personal protective equipment (Tabah et al., 2020). The high job demands faced by healthcare professionals during COVID-19 have been shown to result in emotional exhaustion and stress (Moreno-Jiménez et al., 2021), and consequently high levels of burnout (Huo et al., 2021).

In the United Kingdom, qualitative research amongst frontline healthcare professionals suggests there have been a number of major challenges faced by healthcare professionals during the COVID-19 pandemic, including a lack of PPE, inadequate training, a lack of consistent guidelines with respect to caring for patients during the pandemic, and a changing and challenging work environment (Al-Ghunaim et al., 2021; Hoernke et al., 2021). Findings from a longitudinal survey of frontline healthcare professionals (specifically nursing and midwifery staff) showed high levels of negative psychological effects because of COVID-19, including concerning prevalence of post-traumatic stress disorder, stress, and anxiety (Couper et al., 2022).

International efforts have been made to develop appropriate interventions to support the mental health needs of healthcare professionals. Examples have included: stress reduction techniques (Callus et al., 2020), developing effective communication channels between all healthcare staff members (Shanafelt et al., 2020), and more broadly providing appropriate mental health services for healthcare professionals (Sultana et al., 2020). Research in the United Kingdom has included the development of digital packages to support healthcare professionals’ psychological wellbeing (Blake et al., 2020). However, little is currently known about the barriers and enablers to accessing support services, particularly in relation to services that were set up in response to COVID-19. This insight is crucial in order to understand and promote greater uptake of support services for healthcare professionals when working under psychologically challenging circumstances.

Interventions that can be delivered under pandemic conditions, including personalised psychological approaches to supporting mental health and well-being are key to addressing the impacts of COVID-19 (Hogan and Broadcaster, 2020; Holmes et al., 2020; Shanafelt et al., 2020). Research conducted during previous pandemics identified organisational level factors as important in addressing the psychological needs of healthcare professionals (Maunder et al., 2008), such as providing psychosocial support and interventions for healthcare professionals (Chan and Huak, 2004), and clear communication of precautionary measures to help reduce the psychological impact of working during a pandemic (Chan and Huak, 2004).

One of the largest investments the UK government has made in staff wellbeing during the COVID-19 has been the creation of 40 regional dedicated staff support mental health hubs (West Yorkshire and Harrogate Health and Care Partnership, 2021). One of these is the West Yorkshire (WY) Hub which supports over 100,000 staff including those based in the National Health Service (NHS) in the United Kingdom, social care and voluntary sector. The Hub delivers services based on a four-level framework (see Appendix A). Level 1 of the WY Hub services is prevention focused, and involves interventions and measures designed to support (1) a positive staff culture which engenders wellbeing and help-seeking and (2) the embedding of formal and informal structures to ensure that all teams and individuals can access mental health focused conversations to support their wellbeing. Despite the wide range of services offered by the WY Hub, many staff do not engage with the support which it offers despite reporting high levels of mental distress. The Hub covers 140,000 staff and volunteers, and data collected between July 2021and February 2022 shows a very low number of referrals for psychological support (*n* = 450 referrals [0.3% of eligible staff]; Hinsby et al., 2022). Understanding the perceived challenges in accessing the WY Hub may help to inform implementation of the hub services more broadly, consequently leading to greater uptake of services.

Consequently, there is an urgent need to understand the barriers and enablers to accessing services aimed at supporting healthcare professionals’ mental health and wellbeing needs. Previous research suggests that employees are less likely to use workplace counselling services when their organisation is: perceived as psychologically unsafe (Athanasiades et al., 2008), if they suspect it is not confidential (French et al., 1997; Athanasiades et al., 2008), or that their organisation is only offering the services to avoid litigation (Lockwood et al., 2017). Despite experiencing elevated levels of mental distress since the onset of the pandemic, engagement with support services remains lower than expected, suggesting an urgent need to examine this further.

In order to address this gap in the literature, the present study reports the results from a qualitative study examining the barriers and enablers for healthcare professionals accessing the WY Staff Wellbeing Hub.

## Materials and methods

### Design and sample

We conducted a qualitative study using semi-structured interviews, to explore the barriers and enablers of healthcare, social care and voluntary, community and social enterprise (VCSE) staff accessing the services offered by the staff wellbeing hub. In order to obtain the most detailed insight into the barriers and enablers to accessing the WY Staff Wellbeing Hub, we used a combination of convenience and purposive sampling for participants who had and had not accessed hub services. Our sampling frame included healthcare professionals who worked for the NHS, social care and voluntary, community and social enterprise (VCSE) within one integrated care system in the North of England. We included staff and volunteers within any service and any role and did not restrict to clinical or patient-facing roles.

Semi-structured interviews were selected for their flexibility, which allows participants to direct the interview process while still responding to questions in specific areas. Interviews were conducted by one of the authors (LP; a researcher trained in qualitative methods) using a topic guide (presented in Appendix B) that probed participants’ experiences of accessing the WY Hub services. The choice of one-to-one format enabled participants to explore potentially sensitive topics and recount their histories in depth to the degree they preferred, without any peer influence or concerns regarding confidentiality (Sim and Waterfield, 2019). Remote interviews were chosen for this study as this was thought to be more convenient for professionals, considering their busy schedules. Data collection ceased at the point of saturation, where the research team agreed by consensus that no new themes were emerging from the data.

### Procedure

Ethical approval for the study was obtained from the School of Psychology, University of Leeds ethics committee (Ref: PSYC-277; Approval date: 08/06/2021). Participants were recruited *via* posters and messages circulated *via* organisation-wide emails and the organisational social media accounts. Participants were interviewed remotely and interviews were recorded and transcribed verbatim *via* Microsoft Teams. Participants who had accessed the hub services (*n* = 10) were recruited using a convenience sampling approach; thus, if the participants completed their Level 4 intervention and were interested in participating in the study, their details were shared with the research team through the WY Hub. No participants who had accessed Level 4 services were excluded. Participants who had not accessed the hub services (*n* = 15) were recruited *via* a purposive sampling approach, which involved recruiting individuals who had not used WY Hub services. Staff were recruited through advertisements and excluded those who had used WY Hub services. Demographic data were collected (gender, ethnicity, age, occupational group and sector and job role). Age was recorded in 10-year age bracket categories.

### Data analysis

A reflexive thematic analysis was used as the analytical approach (Braun and Clarke, 2019). This approach required following the six steps outlined by Braun and Clarke (Braun and Clarke, 2006). The first step is familiarisation with the data before coding could occur. For coding data analysis, a manual approach was used and utilised an inductive analysis technique. During the analysis, an open approach was used during the initial coding, which helped ensure both the codes and the themes were grounded in the data. The codes and themes were initiated without distinguishing between enablers and barriers. A semantic level was undertaken for coding, covering what was explicitly expressed by participants. The level of analysis progressed, using theory, from description to interpretation and clarified the health care staff’s barriers and enablers. Next, codes were grouped into themes. Each theme represents a “central health care profession concept” describing a meaningful pattern in the data (Braun and Clarke, 2019). AA created the themes, which LP verified. The data analysed was in relation to the self-reported personal experiences and views of healthcare professionals.

## Results

Twenty-five participants took part, including 23 women and 2 men who had a mode age category of 31–40. Participants were from a variety of different organisational backgrounds including healthcare, social care and voluntary, community and social enterprise (VCSE) sectors. Participant characteristics are presented in Table 1. Participants’ experiences of accessing the WY Hub support services were captured in four key themes, (1) *Environment and Atmosphere in the Workplace*; (2) *The Impacts of COVID-19*; (3) *Confidentiality*; and (4) *Awareness and Communication of Resources*. Illustrative quotes are provided verbatim to illustrate themes, with participant ID number displayed in parentheses.

**Table 1:** Participant demographics (*n* = 25).

Gender	n (s3)	n (s1)	N (total)
Male	2	0	2
Female	13	10	3
Age			
Under 20			
21–30	4	3	7
31–40	4	4	8
41–50	3	0	3
51–60	4	3	7
61–70	0	0	0
>70	0	0	0
Ethnicity			
White	12	9	19
Black or Black British	1	0	1
Asian or Asian British	1	0	1
Mixed	0	1	1
Other	1	0	1
Setting currently working in			
NHS	10	8	18
Voluntary	2	2	4
Social care	3	0	3
Role			
Wellbeing worker	1	0	1
Project manager	1	0	1
Caregiver	1	0	1
Nurse	3	2	5
System engagement Coordinator	1	0	1
Matron Learning Disability Service	1	0	1
Care worker	1	0	1
Quality and patient safety lead	1	0	1
Business support officer	1	0	1
Doctor	1	0	1
Assistant Psychologist	1	0	1
Communications	1	0	1
Support worker	1	0	1
Service Director	0	2	2
Project support	0	1	1
Manager	0	3	3
Business services coordinator	0	1	1
Community development practitioner	0	1	1

### Theme 1: Environment and atmosphere in the workplace

A primary concern and enabler for hub access was the environment and work atmosphere. This theme had five subthemes: (1) Role of the manager; (2) peer effects; (3) workplace discussions; (4) role of the healthcare professionals; and (5) regulations and policies.

#### Role of the manager

Participants reported that managers could support workers and facilitate access to mental health services, but their ability to do so required a visible presence in the workplace and a knowledge of what is available.

"She [manager] was happy to read [about an underlying health condition] and that was useful and trying to adapt where you know and refer me to occupational health where possible. So I think she's been really flexible and done what she can do within the context of yeah the current environment."[P5, accessed services].

When a manager was not visible, fewer people asked for their help, creating a work environment that discouraged asking for assistance. It was perceived that managers could often underestimate the need for services and this, coupled with a lack of awareness about services, may have negative consequences for workers.

“She[manager] had the support she needed to support me directly or indirectly, but I don’t feel that I was supported.” [P3, did not access services].

"Organisations that have third sector organisation managers who have different targets and different ethos. So contacting XXX NHS trust manager and they said all these things that you could do this and that. The third sector organisation don't have that support system, have no knowledge or other support system in general. And they kind of just discredited it. So it just seemed completely.. Pointless asking for help because nothing was done." [P6, did not access services].

Developing a culture of workplace support was perceived to involve the worker, their direct manager and higher-level executives. In order to be of benefit to their team, participants reported that a manager must also look after their own mental health. A manager sharing some of his or her own experiences around accessing mental health services was thought to send a positive message to workers, particularly new employees.

“Now that was a big hurdle to overcome, but I was helped in that by other senior people. Sort of like saying it's all right to seek help for these things, including our chief executive and you know my line managers and executive director,… so I was helped in that by their example.” [P3, accessed services].

#### Peer effect

Some participants from the healthcare sector reported experiencing stigma around accessing mental health services.

“I think it sometimes makes it harder. Because amongst colleagues, particularly, there’s still that stigma.” [P3, did not access services].

Therefore, peer support was perceived to be essential for health workers seeking this kind of assistance; seeing a colleague reach out enabled others to reach out, especially if the colleague’s experience was positive. Peers also engendered confidence in the Hub and other mental health services. Health workers who worked within the mental health sector found there was less pressure to keep mental health concerns private.

“At the moment I feel that I'm struggling. I think …I'm actually one of the lucky ones that I feel quite well supported by my team and by my consultants.” [P8, did not access services].

“I think teams would report that they feel supported by their immediate team, probably better than anyone else….”[P7, did not access services].

#### Workplace discussions

Participants believed that discussions were a tool that could be used as a way of increasing awareness of the Hub, and recommending Hub services to others. Discussion was believed to help create an environment of consent, ease stigma and build a solid ground for accessing care. Workplace discussions on mental health could be both formal or informal. More informal discussions were described as including a focus on personal experiences in overcoming barriers to accessing mental health services. More formal discussions did not focus on personal experiences, but instead emphasised mental health more generally.

“I remember, you know, support is available. If anyone is struggling, we mention that with the person.”[P4, accessed services].

“Can be open about their own experiences and would be a real benefit and you know encourage or enable other people to access the part if they needed it” [P1, did not access services].

“I think other people having positive conversations about it's a big one. And if you hear somebody had a positive experience, I think you you're more likely to feel able..” [P12, did not access services].

‘Indirect’ discussions about mental health were also perceived to be important, as they led the worker to sense that there was a level of support in the workplace around mental health concerns; workers ‘tested’ whether they were in an accepting environment through discussion, and the depth this acceptance. This then led them to have the confidence to speak up about any mental health concerns they may have had.

“How are you? How was your weekend kinda thing and it's smart? It's smart, focused on work though, rather than formally being, uh, just about well being” [P3, did not access services].

“Acceptable, so more people say I'm struggling and this is what I want. This is what I'm struggling with. I think it makes it more OK. Like, um, you know, if you have a colleague that says or you know I've struggled with depression. It really breaks down those barriers.” [P8, did not access services].

#### Role of the healthcare professionals

Workers within the healthcare system included healthcare providers, social workers, administrators and volunteers. Depending on the person’s attitude and the work environment, working within this sector could either facilitate or hinder access to care. A healthcare worker’s ability to recognize their need for mental health care, seek help and accept care varied depending on their personal beliefs and the work environment. Participants believed that healthcare workers tended to wait until they were at breaking point to ask for care as they believe that there are people more in need than themselves.

“Well, I think that's a major issue is that people aren't taking care of themselves because actually their focus has always been on taking care of other people or doing things that are providing other people care.” [P3, accessed services].

“It may be that they end up having to go off sick. I don't know. But um, I think people I've seen people wait until they just get burned out and then they just said that, you know, they have to receive support.” [P8, did not access services].

Stigma may exist around healthcare workers accessing mental health services; a personal experience of having a mental health issue and being a user of services can reduce a worker’s sense of this. Being a caregiver and a receiver simultaneously can be challenging, depending on the severity of the case and the person’s issues, and whether they are a barrier to continuing to work. Being a manager posed further challenges, as the expectation was that they could cope with difficulties.

“If somebody is going through a hard time emotionally, I think there's still a stigma is it's. It's a wider society thing, but I think it's still.” [P8, did not access services].

“I'm very senior, so there's the idea that I should be able to cope. I'm helping my staff cope, but I should be able to do it so that and that's a personal.” [P3, accessed services].

#### Regulations and policies

Some workplace policies were perceived to be barriers to accessing care, and participants believed these needed to be changed or made more flexible. The steps that policies stipulated should to be taken were described as often overly complex and discouraging to workers seeking help. Knowing that there were inadequate policies around mental health suggested to workers a general lack of interest in their well-being, and negatively impacted on their decision to seek help.

“You know, if their policy is to look after this stuff] supporting policies[. I know I said about getting a balance, I said, but I think you know not even a card or flower or you know anybody else that you would think that it's a little while.” [P3, did not access services].

“Some webinar and you had to put your line managers email address in to get them to sign off that you could go]policies to access services[. So things like that would put me off.” [P5, did not access services].

### Theme 2: The continuing impact of COVID-19

This theme captured the impacts of the COVID-19 pandemic, which has led to changes in views on mental health and its importance. This theme had three subthemes: (1) stigma (attitudes towards mental health); (2) mental health challenges; and (3) workload and pressure.

#### Stigma (attitudes towards mental health)

Stigma was an issue long before the pandemic, but participants reported that pandemic conditions may have changed stigma-related attitudes. It was believed that the pandemic pressures opened up discussion around the need for staff to access care and led to a greater level of management understanding.

“I think, particularly managers, I've had a few line managers since I started, and the CEO seemed to be much more concerned with staff well being than they were before [the pandemic].”. [P5, did not access services].

“Prior to the pandemic, I would not have found those things] available support and services[, so actually, I think it’s been a benefit in some respects.” [P11, did not access services].

“I know that I would certainly be a bit more inclined to do that now] accessing services after the pandemic started[, and I’d feel a bit more comfortable.” [P3, did not access services].

However, it was believed that stigma still remained, even if it was not overly apparent. This was especially true in the case of workers with previou+++s mental health issues.

“I think overall it's a very positive reflection] work environment after the pandemic[. I think there's a lot of understanding, and I think there's a lot of, um, even attempt to really address that and talk about it. Destigmatize make sure that there's access to support, but I think there is still a little bit of a lack of understanding, particularly from the sort of senior leadership when they’re not from a mental health background.” [P10, accessed services].

#### Mental health challenges

The impact on workers of the mental health challenges presented by COVID-19 was perceived to be both positive and negative. Well-being was perceived to be dramatically affected, and it was felt that the support which was on offer had not caught up to facilitate help seeking. The challenge was more significant for those who have had direct contact with patients as this felt more personally threatening for their health. The impact of this close-contact had left its mark for longer than expected, and was especially damaging when support was lacking.

“The lockdown has a mass effect on everyone, but at the same time, I don’t think that they realise how much it has affected individual members of staff.” [P3, did not access services].

“I think it's probably been different for different groups, and the areas that I'm manage, um, a predominantly in patient areas, so it's tougher been working throughout the pandemic” [P7, did not access services].

]"Covid19 effect[Effecting workflow and people are struggling in general. I think that it just as a lot of people going up work-based stress, a lot of people aren't feeling supported. A lot of people are leaving. There's really high turnover of staff." [P6, did not access services].

However, support and compassion were perceived to have increased overall, which benefited help-seeking. Similarly, the support and encouragement for accessing the service changed in a positive direction which both became an enabler.

“I think it’s opened up that call to talk more openly] work during COVID19[, check in with each other, and try to be a bit kinder to each other.” [P4, accessed services].

#### Workload and pressure

Workload and pressure resulting from the pandemic were perceived to have a significant impact, and were reasons to both access and not access the Hub. The pandemic impacted workloads greatly, but support and management had not matched up with this.

“I think the workloads have increased significantly, but the staffing and especially and management level hasn't. So it has felt like we've been on our knees for a very long time, and so there's a level of just sustained exhaustion across the team at the moment.” [P10, accessed services].

Healthcare professionals felt the need to cope with difficulty because of the nature of their work and COVID restrictions, and reported they had forgotten to take care of themselves. Working with patients with COVID had put extra pressure on them, yet the desire to be productive had led them to work excessively.

“It’s just about the ongoing setup of the pandemic. For example, burning out because they’re trying to do everything and then doing COVID stuff on top.” [P2, accessed services].

“I think you can get to a point where you you're so overworked….

It got so much on that you don't feel that you have the time for self care.” [P3, accessed services].

Workload and life pressure impacted people seeking help and became a barrier. However, in a supportive environment, the pandemic burden was perceived to trigger a ‘tipping point’ of widespread staff stress which acted as a potential catalyst for help-seeking.

### Theme 3: Confidentiality

Concerns about privacy and confidentiality were both a barrier and enabler to accessing Hub services. This theme had three subthemes: (1) confidentiality; (2) personal factors; and (3) freedom to speak.

#### Confidentiality

Knowing that their information and sessions were confidential was important to help-seekers and service users.

"I think something could.. have to be anonymous, that isn't directly connected to work. I think it would make a big difference." [P6, did not access services].

Participants saw confidentiality as a key concern when seeking and accessing support services. The importance of confidentiality was recognised by several participants, including those who had not accessed the Hub services.

“Well, I just probably reiterate if I think someone going through a hard time that the Hub is there for them. And if they don't want to disclose obviously to their Trust and occupational health. And then you know that is there for them as a different route to go down.” [P7, accessed services].

“I would recommend it] the Hub services[to people that I manage. It is external, so people feel a little bit more relief.” [P4, accessed services].

Services similar to the hub were believed to be available but avoided due to possible confidentiality breaches. Participants reported that potentially knowing other colleagues in the same support groups could add as a barrier to engaging in mental health support services. Thus, the Hub and other services were preferred.

“If I’m referred to a group kind of therapy, I can’t do a group, because I might end up with some service users who I have worked with or will work with, so that’s a real challenge.” [P5, accessed services].

“Getting help can be challenging when you work in the field because you know people normally. There’s a lot of barriers.” [P5, accessed services].

#### Personal factors

Personal factors were highlighted as potential barriers to accessing internal mental health support services. Some participants reported difficulties in acknowledging the need for mental health support, whilst others reported the stigma associated with accessing mental health services may act as a barrier to engaging with services. Thus participants expressed a preference for the Hub, rather than services that were connected with their own place of work.

“So I perhaps we find it quite difficult to accept that I needed help, and I think because of that I made, I got myself into a much worse place.” [P8, accessed services].

“I don’t want anybody at work to know, but that’s more of a personal thing.”[P2, accessed services].

“I’ll reiterate that the barrier to people not accessing these services is themselves.” [P11, did not access services].

Personal issues linked to previous experiences of accessing mental health support services were described by participants. Preconceptions of mental health services were also perceived as a potential barrier to finding and accessing support services. One of the personal enablers to accessing the Hub was a willingness to receive care, and to act as a role model for others who would also benefit from mental health support services.

“I think they're still barrier, because basically I mean. I didn't know about It, when I do know the places are fully booked.” [P3, did not access services].

“I think the most difficult thing is acceptance in yourself that you can allow yourself to receive care.” [P6, did not access services].

“*From what I’m seeing, I don’t think people are going to survive]to access the services[if we don’t.” [P5, accessed services]*.

#### Freedom to speak

Fear of speaking freely when receiving psychological support, due to being based in shared work environments, could be a barrier and limit the potential benefits of the Hub and other services.

“*I can’t work in the office without actually having to find a room to sit by myself, because of the nature of my meeting.” [P11, did not access services]*.

A similar issue, especially with working at home, was the lack of a private place to conduct sessions and share personal stories. This issue was linked to the limited time available for sessions, which might affect user privacy.

“Feeling like I had no private space. In my own home, it was more problematic, feeling like I could be overheard.” [P5, accessed services].

### Theme 4: Awareness and communication of resources

This theme captured the importance of communication methods and how these could be both significant barriers and enablers to accessing the Hub. Findings indicated that messaging and communication about the Hub and other services should be clear as this may increase user acceptance and utilisation. It was reported that it was critical to use a clear and realistic message about the care available and present the concept in an interesting way.

"I know of it [WY Hub], but I don't know really anything it does to be honest." [P6, did not access services].

It was also essential to understand the potential barriers to reaching target audiences and choosing appropriate methods to combat this. One barrier, linked to the comment above, was a lack of promotion of the service, such that some participants reported forgeting it exists. Another barrier described by some participants was a lack of technological knowledge, which meant they did not optimally utilise online information and digital communications.

“It’s really hard to know what to click on, so just make it as simple as possible. Just the information.. really straightforward, call this number.” [P5, accessed services].

“I have an organisational [third sector employee] email and an NHS email, and it goes to your NHS email, and some people don’t access that email.” [P11, did not access services].

Different means of communicating about the hub and other services was perceived to have differential effects. One-to-one interaction was perceived to be potentially effective, as were web pages detailing the features of the Hub. Participants reported that extra resources and support material may facilitate access to the Hub and serve the Hub’s aims. Whatever method was chosen, participants suggested that it needed to be clear and direct.

“People went, Oh yeah, I forgot about that [Hub resources] People go blind when it’s out there all the time and forget about it.” [P11, did not access services].

## Discussion

### Principal findings

This study examined the barriers and enablers to accessing a staff mental health and wellbeing hub, set up in response to the challenges faced by healthcare professionals as a result of the COVID-19 pandemic. There were four important findings of the present research, which could be used to increase uptake and engagement with the Hub. First, organisational environments were perceived as an important enabler of accessing the hub services for mental health and wellbeing support. Second, organisations recognising and responding to the ongoing pressures of COVID-19- specific challenges were perceived to create a culture more conducive to help-seeking for mental health more broadly. Third, confidentiality was perceived to be a crucial enabler of accessing mental health services; organisations should recognise individual aspects of confidentiality, including any personal factors which may prevent them from accessing services. Fourth, clear and consistent communication of the benefits of the Hub may be a way of encouraging help-seeking for mental health challenges among healthcare professionals.

### Comparison with existing literature

There are promising findings that organisational factors and the healthcare environment may be an important enabler of encouraging healthcare professionals to seek help for mental health challenges faced during the COVID-19 pandemic (Chan and Huak, 2004; Maunder et al., 2008; Shanafelt et al., 2020). Our findings extend the existing literature by suggesting that having the support of managers and other staff members may also be crucial in facilitating access to mental health services. With respect to accessing the Hub services in the present study, our findings also identified a key role of positive discussions between staff in encouraging and enabling access to the service.

The recognition of the COVID-19-specific physical and mental health challenges (such as increased workload and the mental health burden of working during a pandemic) were recognised by participants, and is consistent with the broader literature (Cai et al., 2020; Al-Ghunaim et al., 2021; Hoernke et al., 2021; Couper et al., 2022). Recognising and reducing such pressures may be an important enabler to staff accessing mental health and wellbeing services more broadly, as well as reducing the stigma often associated with help-seeking amongst healthcare professionals (Adams et al., 2009; Wallace, 2012), where staff may sometimes be discouraged from talking openly or seeking help for psychological problems (Adams et al., 2009; Ross and Goldner, 2009). Research also suggests an environment with unclear and changing COVID-19-related guidelines may create an additional psychological burden for healthcare professionals (Jessop et al., 2020). Whilst our findings suggested that whilst such pressures had a negative impact on mental health of staff, they also acted as an enabler for accessing the Hub by creating a more open culture around mental health, which served to reduce stigma.

The importance of clear communication of mental health services available for healthcare professionals was highlighted as an important enabler of uptake of the Hub. User acceptance of the hub could be further strengthened with greater emphasis on communicating the benefits of the Hub, as a way of encouraging people to seek help for mental health challenges. Our findings highlight the potentially important role of training for healthcare providers to raise awareness of the stigma sometimes associated with mental health amongst healthcare professionals (Ungar et al., 2016).

### Implications

Health, social care and VSCE professionals are facing considerable mental health challenges, which have been exacerbated due to additional pressures because of the COVID-19 pandemic. Our findings suggest that there are specific steps that can be taken to promote greater uptake and engagement with services more broadly, aiming to support the mental health and wellbeing of healthcare professionals. In particular, organisations should (1) provide regular and ongoing email communications about the services which are on offer to their staff and ensure these are going to the most appropriate email inbox for staff; (2) provide training and information for managers in order that they can best support the staff they manage; (3) provide confidentiality for staff accessing and using these services and highlight this in all communications and (4) work towards developing a more open culture around mental health. These recommendations are based on findings regarding access to one UK staff Mental Health and wellbeing Hub. However, they have relevance for both the remaining 39 UK Hubs and also for other occupational services providing mental health support within the United Kingdom and internationally.

Culture change is challenging and complex (Scott et al., 2003; Carroll and Quijada, 2004), and our findings can be regarded as the start of a wider investigation of the needs of healthcare professionals with respect to supporting mental health and wellbeing. Future research should aim to examine changes in uptake of mental health support services as a result of interventions targeting: (1) increased organisational awareness and support for mental health support amongst healthcare professionals, (2) increased organisational response to COVID-19-specific challenges, and (3) more effective communication of the benefits of support services for healthcare professionals. It would also be valuable to examine whether access to mental health services differs according to sociodemographic factors. This research will help ascertain which barriers and enablers should be prioritised as targets when aiming to improve service uptake.

### Strengths and limitations

The study benefited from an adequate sample size and a purposive sampling approach, which aimed to ensure that the views of diverse staff were sampled, including those from a range of genders, ethnicities, professional background and various organisations. However there are limitations to the study. Whilst national workforce data suggests that the health and social care workforce is dominated by women (Boniol et al., 2019), our sample was more heavily weighted in favour of women compared with national data (92% in our sample versus 70% in national data). Whilst our sample included people who did and did not engage with hub, it may be possible that additional barriers and enablers may have been captured using a theoretical framework focused on implementation. Whilst using a reflexive thematic analysis allowed important themes to be identified from the data, future research may benefit from using specific frameworks to identify specific targets for interventions focused on improving the implementation of interventions in healthcare settings (Murray et al., 2010; Atkins et al., 2017).

## Conclusion

The mental health impacts of the COVID-19 pandemic on healthcare professionals have been significant. Whilst mental health services are currently being developed to support healthcare professionals, recognising the barriers to accessing help, and responding to the enablers to accessing services is a crucial first step in promoting greater uptake of services. A more proactive approach to supporting healthcare professionals who are facing psychological challenges could be adopted by healthcare organisations. Consequently, greater uptake of services promoting health and wellbeing is an important step in supporting healthcare professionals in reducing the psychological challenges faced during times of increased pressures, and encouraging more focused conversations to support healthcare professionals’ wellbeing.

## Data availability statement

The raw data supporting the conclusions of this article will be made available by the authors, without undue reservation.

## Ethics statement

The studies involving human participants were reviewed and approved by School of Psychology, University of Leeds ethics committee. The patients/participants provided their written informed consent to participate in this study.

## Author contributions

CK, LP, and JJ conceived and designed the study. LP collected the data. CK, JJ, AA, and LP conducted the analysis. All authors helped to interpret the data. CK drafted the article. All authors contributed to the article and approved the submitted version.

## Funding

The research was funded by a grant from the West Yorkshire Health and Care Partnership.

## Conflict of interest

The authors declare that the research was conducted in the absence of any commercial or financial relationships that could be construed as a potential conflict of interest.

## Publisher’s note

All claims expressed in this article are solely those of the authors and do not necessarily represent those of their affiliated organizations, or those of the publisher, the editors and the reviewers. Any product that may be evaluated in this article, or claim that may be made by its manufacturer, is not guaranteed or endorsed by the publisher.
