# Supplementary material for: Barriers and enablers to accessing support services offered by staff wellbeing hubs: A qualitative study
Source: Front Psychol. 2022 Nov 15;13:1008913. doi: 10.3389/fpsyg.2022.1008913 (PMC9706200; doi:10.3389/fpsyg.2022.1008913)
Supplement: Supplementary file 1 [file Table_1.docx]

1. Is staff wellbeing discussed between staff?
   1. Explore nature of the discussions
   2. Positive/negative/stigma?
   3. Who has these discussions? Between staff, or between staff and managers?
   4. Explore formal/informal nature of discussions – raised in staff meetings? Managerial meetings? Or raised in informal conversations outside of formal settings?
2. Are you aware of any strategies or services offered to support staff wellbeing [focus on services not related to the hub]?
   1. Explore what staff member is aware of
   2. Explore access – do they know how to reach these services?
   3. Explore barriers – reasons why they might not access these services
   4. Explore facilitators – what might make this easier? Probe for examples of services they have offered?
3. The staff wellbeing hub offers more services than 1:1 therapy – are you aware of these services?
   1. Explore knowledge of the hub
4. Do you feel the hub has been a positive change in your organisation?
   1. Explore how it is positive if yes/why it isn’t positive, if it’s not
5. Have you discussed the hub with your colleagues?
   1. Explore whether this has been discussed, or not, and whether the participant has encouraged uptake of hub services by others. Explore why this has/hasn’t been discussed
6. Is your manager aware of the hub?
   1. Explore manager awareness of the hub
   2. Explore manager attitude towards the hub
   3. Explore manager approach to mental wellbeing in general
7. [If the participant is a manager] Would you encourage the staff you manage to use the hub?
   1. Explore reasons – including benefits/barriers/costs

Additional questions for staff who had accessed 1:1 therapy:

1. How did you come to hear about the services offered by the hub?
   1. Explore who/where they learned about the services
   2. Explore first impressions
2. How did you access the hub?
   1. Explore support/encouragement to access the hub
   2. Explore barriers encountered/how these were overcome
