# Supplementary material for: Barriers and enablers to accessing support services offered by staff wellbeing hubs: A qualitative study
Source: Front Psychol. 2022 Nov 15;13:1008913. doi: 10.3389/fpsyg.2022.1008913 (PMC9706200; doi:10.3389/fpsyg.2022.1008913)
Supplement: Supplementary file 2 [file Image_1.pdf]

Hub overview

The West Yorkshire (WY) Staff Wellbeing Hub is one of 40 regional dedicated staff support mental health hubs which were commissioned in January 2021 in a response to the impact of COVID-19 on the workforce. The WY Hub supports over 100,000 staff including those based in the NHS, social care and voluntary sector. The Hub delivers services based on a four-level framework (Figure 1). The first two levels are prevention focused; they involve interventions and measures designed to support 1) a positive staff culture which engenders wellbeing and help-seeking and 2) the embedding of formal and informal structures to ensure that all teams and individuals can access mental health focused conversations to support their wellbeing. Levels 3 and 4 are proactive. Level 3 is focused at the teams level, ensuring that teams impacted by work stressors can access resources to support them and help them recover from the impact of acute stressful events. These teams can also access ongoing support to identify individuals who may need referral for further, individualised interventions. Level 4 is focused at the individual level, enabling the provision of timely access to high-quality, culturally sensitive and evidence-based interventions.

This report has been prepared as a collaboration between the WY Hub and the University of Leeds. It presents a comprehensive evaluation of the hub and its services offered during its initial commissioned phase from January 2021 – March 2022. In particular, this report aims to evaluate 1) access to the hub and 2) experiences and effectiveness of the services it provides. The findings are reported in line with the tier structure presented in Figure 1.

At the time of commissioning the work, the geographical boundaries included Harrogate. However from April 2021 the geographical commissioning boundaries changed and Harrogate moved to a neighbouring ICS. Some of the early data includes Harrogate but the later data excludes Harrogate and represents the current picture.

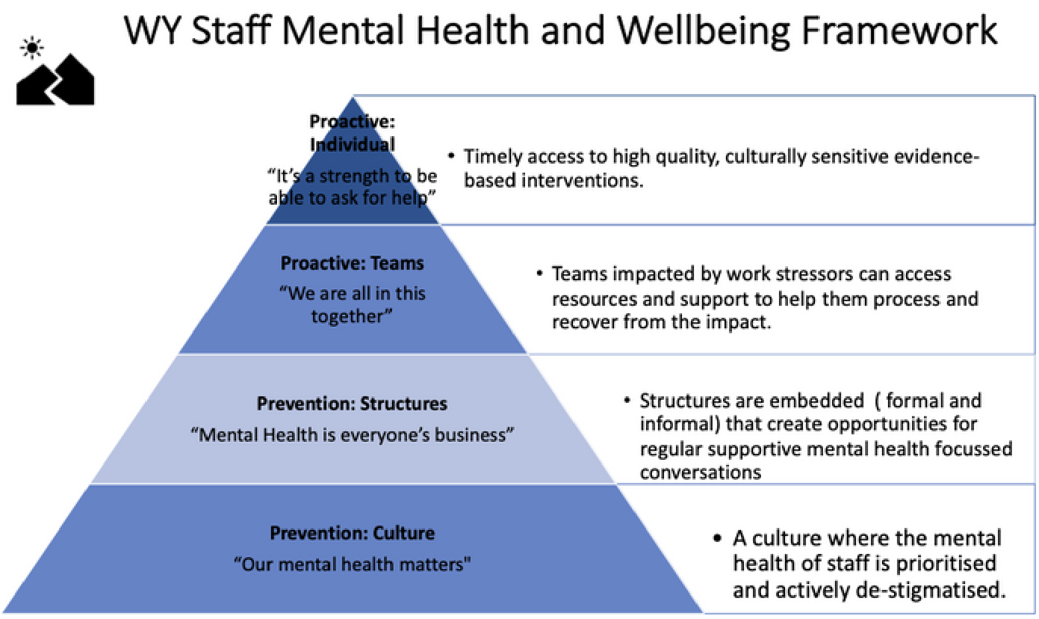

Figure 1
